# Burnout and Cognitive Functioning: Are We Underestimating the Role of Visuospatial Functions?

**DOI:** 10.3389/fpsyt.2022.775606

**Published:** 2022-03-23

**Authors:** Panagiota Koutsimani, Anthony Montgomery

**Affiliations:** Department of Educational & Social Policy, School of Social Sciences, Humanities and Arts, University of Macedonia, Thessaloniki, Greece

**Keywords:** job burnout, cognitive functions, visuospatial functions, healthcare workers (HCW), patient safety, mini-review

## Abstract

Job burnout is a psychological syndrome which results from chronic occupational stress and cognitive impairments are among its negative consequences. The demands of the COVID-19 pandemic have challenged the healthcare system increasing the risk of job burnout among healthcare professionals. The studies conducted so far have mainly focused on the effects of job burnout on executive functions. Visuospatial functions are a cognitive domain which plays an important role in healthcare workers' optimal performance. Healthcare workers are constantly relying on their visuospatial abilities in order to care for their patients as they are required to use techniques that involve manipulation of medical instruments, they need to have excellent hand-eye coordination and great perception of spatial anatomy, factors that can affect healthcare workers' performance is of significance and can put patient safety at risk. However, our understanding of how visuospatial functions are being affected in job burnout is limited. The scope of this mini-review is to examine the evidence concerning the relationship of job burnout with visuospatial functions. The sparsity of the relevant empirical evidence does not allow for definite conclusions. However, given the implications of diminished visuospatial abilities in patient safety we highlight the need for studies exploring the effects of job burnout on visuospatial functions. Limitations of studies are discussed.

## Introduction

Job burnout is common in healthcare (1) affecting not only healthcare professionals' physical and mental health but also patient safety (2). The increasing demands of patient care along with the uncertainty regarding the ways on how to handle the COVID-19 pandemic has led undoubtedly to an increased risk of job burnout. Thus, early detection and treatment of job burnout are of great importance.

Job burnout is a psychological syndrome characterized by three dimensions, namely exhaustion, cynicism and reduced personal efficacy (3). A growing body of research suggests that cognitive impairment is also a prominent job burnout characteristic [e.g., (4, 5)]. Importantly, the relevant prospective studies indicate the long-lasting negative effects of job burnout on cognition (4, 6, 7). Observations that led Desart et al. (8) to propose the inclusion of two additional components in the job burnout definition; emotional and cognitive loss of control.

In their systematic review, Delgikaris et al. (9) argued that executive functions is the cognitive domain that appears to suffers most in burnout. Recent studies also corroborate this observation as they have also detected executive functions deficits amongst burned-out employees (5, 10, 11). Executive functions allow the performance of complex tasks that require skills such as planning, decision making, cognitive flexibility, focused attention, inhibition, and interference control (12). These skills are necessary for healthcare professionals and deficits in this cognitive domain could put at risk patient safety. Even though a considerable amount of evidence supports the adverse effects of job burnout on executive functions, it is of significance to note that executive functions are also the most studied cognitive domains in the job burnout research (9); possibly due to the fact that they have a key-role in employees' efficiency in meeting work demands (e.g., deadlines, making correct decisions under pressure). The studies assessing a broader range of cognitive functions indicate that other cognitive domains are affected in job burnout as well. Jonsdottir et al. (13), for instance, apart from executive functioning deficits also observed impairments in episodic memory and learning in a group of clinical job burnout patients. Memory problems have been observed in more recent studies as well (14).

A cognitive domain that has not been studied extensively is visuospatial functions. Visuospatial functions concern one's ability to mentally visualize objects and spatial relations, and enable us to perceive, synthesize and combine visuospatial relationships (15). Thus, visuospatial functions are those cognitive functions that allow us to navigate through space and interact with the surroundings of our environment. The ability to perceive the relationships between structures as well as the use of surgical instruments is of crucial importance among healthcare workers and especially among medical care professionals (i.e., surgeons, nurses). Healthcare workers are constantly relying on their visuospatial abilities in order to care for their patients as they are required to use techniques that involve manipulation of medical instruments (16), they need to have excellent hand-eye coordination (17) and great perception of spatial anatomy (18). Furthermore, considering the constant technological advances that necessitate the ability of healthcare workers to learn new technical skills, visuospatial abilities can be of importance for acquiring these skills (19). Therefore, the examination of those factors that can affect healthcare workers' optimal visuospatial performance is of significance as it can put patient safety at risk.

Notably, a recent scoping review emphasized the importance of visuospatial abilities in surgical performance. Specifically, from their literature review the authors observed that individuals with greater visuospatial skills (as measured by standardized neuropsychological tests) showed greater surgical performance (either in surgical simulation tasks or in clinical environments) while they were faster learners comparing to their peers with lower visuospatial skills (20). The authors also highlighted the need for further studies examining whether performance on visuospatial cognitive tasks can predict surgical performance in clinical environments (20). Visuospatial functions are of importance in healthcare and deficits in this cognitive domain could have detrimental effects on patient safety.

The effects of job burnout on the performance of healthcare workers have not received adequate attention (21) while the emotional and physical strains that were posed to the healthcare workers during the COVID-19 pandemic potentially has increased the risk of job burnout (22, 23). The aim of the present mini- review is to summarize and discuss the evidence concerning the effects of job burnout on visuospatial skills with the purpose of outlining the implications of diminished visuospatial abilities in healthcare quality; and highlight the need for further research which will help toward the elucidation of the relationship between job burnout and visuospatial functions. Acknowledging the job burnout effects on healthcare professionals this will enable policymakers to develop more focused prevention strategies and targeted intervention programs leading to the advancement of both healthcare quality and patient safety.

## Studies on the Relationship Between Job Burnout and Visuospatial Functions

To the best of the authors' knowledge the number of studies examining the visuospatial skills of burned-out employees is scarce as only five studies were identified (see Supplementary Material for search strategy). Specifically, Morgan et al. (24) examined the effects of job burnout on the visuospatial skills of special operation military personnel in a two-wave study. The results showed that neither exhaustion not cynicism predicted visuospatial abilities. However, greater levels of personal efficacy led to an increase of visuospatial skills. This finding suggests that one's confidence on their abilities to resolve efficiently work-related tasks can enhance their visuospatial functions during stressful situations. Although these results do not reflect an effect of job burnout on visuospatial functions *per se*, they suggest a potential significance of personal efficacy in predicting visuospatial abilities.

In a similar vein, Österberg et al. (25) measured the long-term effects of clinical job burnout in a group of former burnout patients. Even though the participants did not show visuospatial impairments in the baseline assessment, 20 months after their initial sick leave the participants (who were actively working at the time of the follow-up assessment) showed slightly lower visuospatial abilities. The researchers concluded that this observation might not represent a true effect of job burnout on visuospatial skills mainly due to the lack of empirical evidence supporting visuospatial deficits in job burnout. Indeed, one cannot overlook the fact that this observation might reflect a chance finding or that another factor may have mediated this relationship (e.g., visuospatial deficits could constitute a result of sick leave). Nonetheless, we should not ignore the possibility that visuospatial deficits might develop later as job burnout progresses and remain apparent even after recover. The lack of longitudinal studies however, do not allow sufficient evidence to support this argument.

A recent study observed diminished performance on cognitive tasks measuring visuospatial skills among employees who reported high cynicism levels (26). The researchers also observed that the employees who scored high on cynicism showed worse visuospatial performance comparing to the employees who scored high on exhaustion. Job burnout is mainly regarded as an exhaustion disorder and the basic conceptual model holds exhaustion as the first dimension to develop (27). However, more recent research posits cynicism as an early job burnout phase (28). Thus, these results suggest that visuospatial deficits could occur early in job burnout. Nevertheless, the cross-sectional design of the study does not provide information on how the two variables develop over time.

Jonsdottir et al. (13) compared the cognitive performance between job burnout patients and healthy employees by assessing a wide range of cognitive functions, namely processing speed, attention, working memory, learning and episodic memory, executive functions, visuospatial functions, and language. The researchers found that job burnout patients performed worse on tasks tapping executive functions but no differences were noted in the performance between the patient and the healthy group in terms of visuospatial abilities; indicating that job burnout is not associated with visuospatial functions.

Similarly, Sandström et al. (29) assessed for differences in the cognitive performance between a group of chronic job burnout patients and a group of healthy individuals. Interestingly, the findings of the study showed that the job burnout group showed deficits in visuospatial short- and long-term memory but not on visuospatial constructional abilities. Chronic stress can have negative effects on the hippocampal neural activity (30) leading to memory and visuospatial functioning impairments. Thus, although the researchers did not observe deficits in visuospatial skills, their results suggest that chronic job burnout can impair both the short-term and long-term visuospatial memory. Visuospatial short-term memory is concerned with the maintenance and the manipulation of visual and spatial information when this information is not any more available in the environment thus, it is a critical function as it helps to create and maintain a structured portrayal of the visual world (31). Long-term memory is recruited for guiding visuospatial attention in one's environment and hence, enables the detection of scene changes (32). Applied to the healthcare environment, deficits in short- and long-term visuospatial memory of healthcare workers could disrupt their ability to effectively recall crucial information such as performing spatially complex technical skills (e.g., surgical knot tying) as well as their ability to discern alterations in the immediate surroundings (e.g., the location of medical instruments, perception of anatomy changes during surgical operations).

Taken together, few studies provide some—but not robust—support on the associations of job burnout with visuospatial functions. The relevant studies are limited and the existing ones provide mixed results hence, definite conclusions cannot be drawn.

## Limitations of the Reviewed Studies

In this mini-review three studies were identified depicting the significant associations between burnout and visuospatial functions. However, the current evidence cannot be considered vigorous enough and the limitations of the studies further impede the possibility on drawing accurate conclusions. Specifically, Morgan et al. (24) showed the positive effects of personal efficacy on visuospatial functions while Österberg et al. (25) observed lower visuospatial skills in a former group of job burnout patients after their recovery but not on the baseline. Koutsimani et al. (26) found significant associations only between cynicism and visuospatial abilities. Considering that job burnout is an exhaustion disorder one would expect clearer evidence in support of its consequences on visuospatial skills such as a potential negative impact of the exhaustion dimension on this cognitive domain.

One limitation is that of the five studies identified only two (24, 25) examined the association of job burnout with visuospatial functioning longitudinally. Thus, no safe conclusions can be drawn regarding both the causality and the predictive relationship of the two variables. Moreover, the two prospective studies explored the effects of burnout on visuospatial abilities across two different time points. Although two-wave studies provide some insights regarding a studied relationship, the form of change of two variables over time cannot be established by measuring an event at only two time points (33). Therefore, more prospective studies with at least three waves of data would be more suitable to help researchers further understand the relationship between job burnout and visuospatial functions.

The heterogeneity of the studied populations further limits our understanding of the burnout-visuospatial functions association as some studies examine clinical job burnout while others focus on non-clinical job burnout. Taking into consideration the evidence that point to the negative associations between non-clinical job burnout and visuospatial abilities (26) and the fact at non-clinical job burnout levels employees are still able to maintain their job performance by adopting coping strategies (34), the authors emphasize the significance of examining the job burnout effects on employees who are at the initial burnout stages. To the best of our knowledge, the visuospatial abilities of healthcare workers suffering from burnout has not been examined so far.

Another limitation involves the inconsistency among the tools being used for assessing job burnout. The Maslach Burnout Inventory (35), for instance, takes into consideration all three burnout dimensions while other questionnaires, such as the Shirom-Melamed Burnout Questionnaire (SMBQ) (36), assess only the exhaustion dimension. Additionally, although some researchers use the total job burnout scores, others use the scores on each job burnout dimension; and others detect job burnout levels based on clinical interviews. Thus, it is possible that the inconsistencies among the research tools could result in an underestimation of the visuospatial skills in job burnout. A general consensus on the diagnostic job burnout tools is of importance in order to reach to more valid conclusions. In the studies that were identified in this review, Koutsimani et al. (26) and Morgan et al. (24) assessed each job burnout subscale by administering the General Survey version of the MBI (MBI-GS) (37), Jonsdottir et al. (13) used the total job burnout score on the SMBQ; Österberg et al. (25) included patients who were previously diagnosed with job burnout through both clinical interviews and the score on the MBI-GS exhaustion dimension and Sandström et al. (29) assessed job burnout levels via clinical interviews.

## Discussion

The research studies investigating the effects of job burnout on cognitive performance mainly show that executive functions are the most prominent cognitive domain that is being impaired in job burnout (9). However, they are also the most examined ones. Failure to explore for other cognitive domains raises the question on whether executive functions are indeed the cognitive domain that suffers most in job burnout; or if the lack of studies examining a broader range of cognitive domains have led researchers to reach fallacious conclusions. Studies assessing a wide range of cognitive domains on burned-out employees have revealed impairments in other cognitive functions as well (13, 26, 38). A cognitive domain that has been largely overlooked in the relevant literature is visuospatial functions. Visuospatial functions are an important cognitive domain as it allows us to navigate in our environment and to comprehend visuospatial relationships. Thus, impairments in visuospatial abilities can negatively affect not only one's personal life (e.g., driving) but also the job performance of those employees who largely depend on this cognitive domain.

So far, only a limited number of studies has examined the associations between job burnout and visuospatial functions and the existing evidence are not vigorous enough to allow accurate conclusions, emphasizing the need for further investigation. This mini-review identified five studies examining the relationship between job burnout and visuospatial functions providing mixed and unclear support for the relationship between job burnout and visuospatial functions. Specifically, Österberg et al. (25) showed the presence of slight deficits in visuospatial abilities and only after recovery from job burnout. The absence of a respective baseline association and the potential unaccounted factors that could mediate this relationship weaken the evidence in favor of the effects of job burnout on visuospatial functions. Indeed, the researchers also observed a reduced performance on tasks tapping attention. Hence, participants' attention deficits could underlie the observed effects of job burnout on visuospatial functions; i.e., not focusing on the details of the visuospatial task.

Koutsimani et al. (26) showed that cynicism was related to lower visuospatial deficits. Nonetheless, due to the cross-sectional nature of the study causality cannot be inferred. Moreover, even though the researchers examined moderators that might explain this association (i.e., depression, anxiety, perceived family support), they did not account for other potential confounding factors such as daily stress beyond the workplace. The results of Morgan et al. (24) also do not provide direct evidence on the impact of job burnout on visuospatial functions as the researchers found that greater levels of personal efficacy—but not inefficacy—predicted greater visuospatial abilities. Hence, concluding that a negative view of one's work performance can result in diminished visuospatial functions would be inaccurate.

The studies that failed to observe any significant associations between job burnout and visuospatial functions also suffer from limitations that could affect the results. The study of Jonsdottir et al. (13), for instance, was underpowered (*N* = 33) increasing the risk of missing a true effect. Additionally, Sandström et al. (29) examined only women. Moreover, although the researchers did not find deficits in visuospatial constructive and perceptual abilities, they observed impairments in visuospatial short-term and long-term memory. Visuospatial functions are mainly clustered in three components; visual perception, construction, and visual memory (39). Thus, these results could indicate an impact of job burnout in certain visuospatial dimensions. Moreover, a recent three-wave Ph.D., study showed a negative effect of cynicism on visuospatial performance (40), suggesting a potential impact of certain job burnout aspects on visuospatial functions.

Overall, the literature lacks a comprehensive examination on the effects of job burnout to visuospatial functions. Moreover, the lack of a general consensus on the tools assessing burnout and the complexity of visuospatial functions requiring their exhaustive examination in order for all visuospatial aspects to be accounted for emphasize the need for thorough examinations. Importantly, in view of the fact that deficits in the visuospatial skills of healthcare workers can have detrimental effects on patient safety and healthcare quality, investigation of the potential impact of job burnout on visuospatial functions is of significance.

The challenges that healthcare workers face in their everyday clinical practice increase their risk of becoming burned-out, affecting both healthcare quality and patient safety. Integral visuospatial abilities are of significance for patient safety and they can be compromised even at the initial burnout levels. The assessment of visuospatial functions of burned-out healthcare workers and if/ how they are affected from job burnout is a prominent avenue of research which will advance our understanding of the job burnout effects and will help toward the implementation of targeted prevention and intervention strategies.

## Author Contributions

AM conceptualized the research topic, reviewed the initial draft, and suggested changes. PK was responsible for searching for and identifying the studies to be reviewed and prepared the initial draft. AM and PK contributed to the preparation of the final manuscript. Both authors contributed to the article and approved the submitted version.

## Conflict of Interest

The authors declare that the research was conducted in the absence of any commercial or financial relationships that could be construed as a potential conflict of interest.

## Publisher's Note

All claims expressed in this article are solely those of the authors and do not necessarily represent those of their affiliated organizations, or those of the publisher, the editors and the reviewers. Any product that may be evaluated in this article, or claim that may be made by its manufacturer, is not guaranteed or endorsed by the publisher.
