# Supplementary material for: Burnout and Cognitive Functioning: Are We Underestimating the Role of Visuospatial Functions?
Source: Front Psychiatry. 2022 Mar 23;13:775606. doi: 10.3389/fpsyt.2022.775606 (PMC8983930; doi:10.3389/fpsyt.2022.775606)
Supplement: Supplementary file 1 [file Data_Sheet_1.pdf]

Supplementary Material

Search Strategy

To identify the studies examining the relationship between burnout and visuospatial functioning, two search procedures were followed. Initially, we searched the online databases PubMed and SCOPUS. Secondly, we proceeded with a manual scoping of the cited studies from all articles that fitted the eligibility criteria in order to account for the studies that did not appear in the database search. By taking into consideration that visuospatial functions are not the primary cognitive domain that is being examined in the burnout research, in order to optimize the efficiency of the search strategy we used the terms “burnout” and “cognitive functioning”; “burnout” and “cognitive performance”; “burnout” and “cognitive impairment” as search terms to find studies that measured for visuospatial skills along with other cognitive domains. We focused on studies that were published during the last decade (i.e., from January 2011 to August 2021). The eligibility criteria included quantitative studies examining cognitive functions with standardized cognitive batteries, peer-reviewed articles written in the English language and full-text articles. **Figure S1** depicts the selection procedure and study identification based on the Preferred Reporting Items for Systematic Reviews and Meta-analyses (PRISMA) statement (Page et al., 2021).

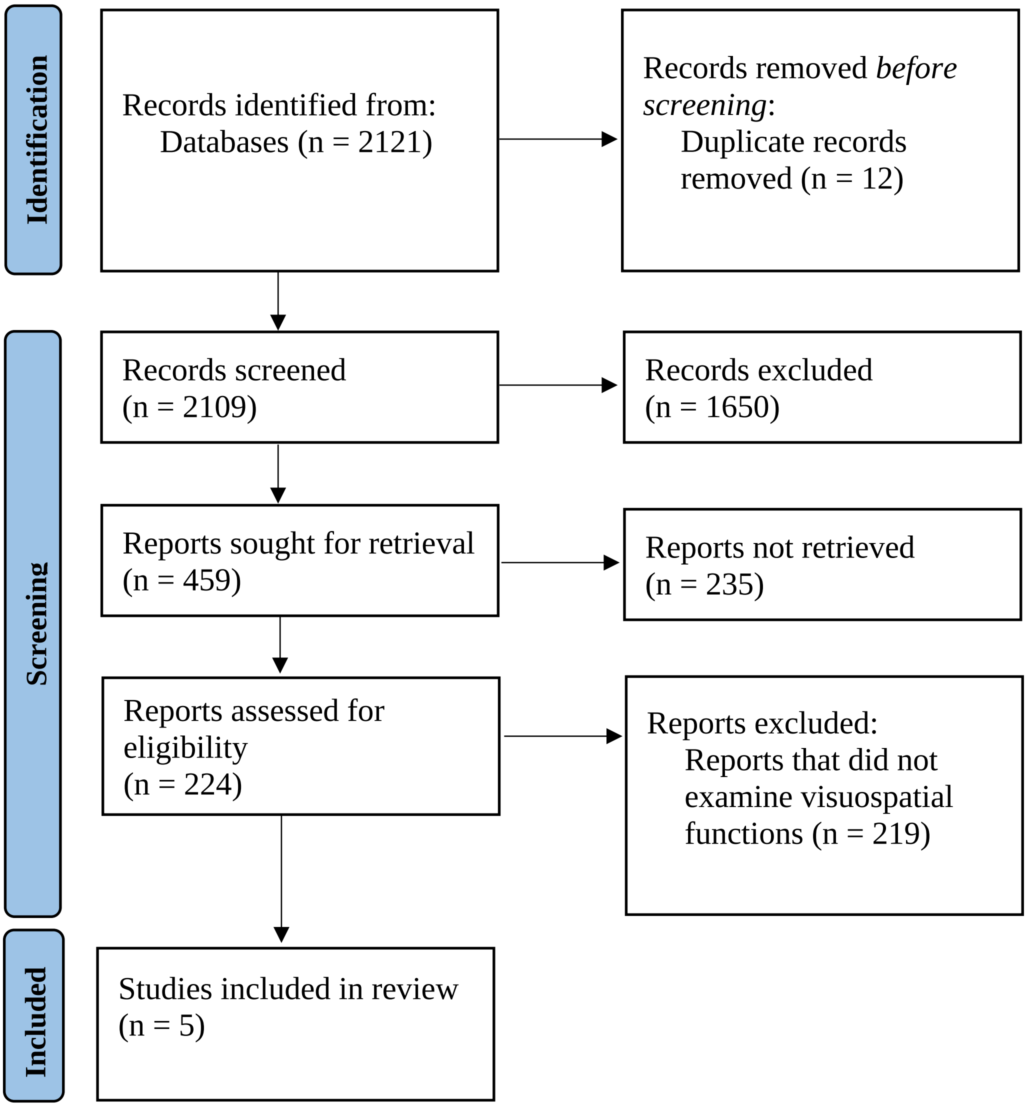

Supplementary Figure S1. Flow diagram for identification of studies examining burnout and visuospatial functions

References

Page, M. J., McKenzie, J. E., Bossuyt, P. M., Boutron, I., Hoffmann, T. C., Mulrow, C. D., Shamseer, L., Tetzlaff, J. M., Akl, E. A., Brennan, S. E., Chou, R., Glanville, J., Grimshaw, J. M., Hróbjartsson, A., Lalu, M. M., Li, T., Loder, E. W., Mayo-Wilson, E., McDonald, S., … Moher, D. (2021). The PRISMA 2020 statement: An updated guideline for reporting systematic reviews. *BMJ, 372*. https://doi.org/10.1136/bmj.n71
